# Modulations of cell cycle checkpoints during HCV associated disease

**DOI:** 10.1186/1471-2334-9-125

**Published:** 2009-08-10

**Authors:** Saira Sarfraz, Saeed Hamid, Syed Ali, Wasim Jafri, Anwar A Siddiqui

**Affiliations:** 1 Department of Biological and Biomedical Sciences, Aga Khan University, Karachi, Pakistan; 2 Department of Medicine, Aga Khan University, Karachi, Pakistan

## Abstract

**Background:**

Impaired proliferation of hepatocytes has been reported in chronic Hepatitis C virus infection. Considering the fundamental role played by cell cycle proteins in controlling cell proliferation, altered regulation of these proteins could significantly contribute to HCV disease progression and subsequent hepatocellular carcinoma (HCC). This study aimed to identify the alterations in cell cycle genes expression with respect to early and advanced disease of chronic HCV infection.

**Methods:**

Using freshly frozen liver biopsies, mRNA levels of 84 cell cycle genes in pooled RNA samples from patients with early or advanced fibrosis of chronic HCV infection were studied. To associate mRNA levels with respective protein levels, four genes (p27, p15, KNTC1 and MAD2L1) with significant changes in mRNA levels (> 2-fold, p-value < 0.05) were selected, and their protein expressions were examined in the liver biopsies of 38 chronic hepatitis C patients.

**Results:**

In the early fibrosis group, increased mRNA levels of cell proliferation genes as well as cell cycle inhibitor genes were observed. In the advanced fibrosis group, DNA damage response genes were up-regulated while those associated with chromosomal stability were down-regulated. Increased expression of CDK inhibitor protein p27 was consistent with its mRNA level detected in early group while the same was found to be negatively associated with liver fibrosis. CDK inhibitor protein p15 was highly expressed in both early and advanced group, but showed no correlation with fibrosis. Among the mitotic checkpoint regulators, expression of KNTC1 was significantly reduced in advanced group while MAD2L1 showed a non-significant decrease.

**Conclusion:**

Collectively these results are suggestive of a disrupted cell cycle regulation in HCV-infected liver. The information presented here highlights the potential of identified proteins as predictive factors to identify patients with high risk of cell transformation and HCC development.

## Background

Infection with HCV accounts for a vast majority of viral hepatitis cases. A recent report suggests its prevalence in geographically diverse areas, affecting around 130 million people worldwide [1]. In Pakistan, the frequency of HCV infection is estimated to be around 6% in general population [2]. These figures are alarming, as patients currently asymptomatic with relatively mild disease may eventually progress to complications of chronic liver disease like, cirrhosis, end-stage liver disease and hepatocellular carcinoma (HCC). According to a recent review published by Raza et al., 45% cases of HCC in Pakistan were found to be positive for HCV antibody [3]. Epidemiological and clinical studies have also demonstrated a causative role of HCV infection in the development of HCC [4], albeit the underlying mechanism is not fully known.

Liver injury is generally believed to be initiated by the death of infected cells afflicting problems of inflammation, regenerative hepatocyte proliferation and fibrosis in the surroundings [5]. In chronic HCV infection, rounds of hepatic cell destruction and regeneration along with fibrosis occur as a result of persistent inflammation. This phenomenon provides the pathogenic basis of HCV associated liver disease. Together, these events keep the dividing hepatocytes susceptible to cellular insult and hence putting them at a greater risk of acquiring mutations.

The molecular events during proliferation are tightly controlled by the cell cycle regulators. During the cell cycle, flawless DNA replication and chromosome segregation are achieved by means of quality control checkpoints that are active at each of the four phases of cell cycle namely Gap1 (G1), DNA synthesis (S), Gap2 (G2) and Mitosis (M). Progression through the phases of cell cycle is driven by temporal activation of specific protein kinase complexes consisting of cyclins and cyclin dependent kinases (CDKs) [6]. In response to cellular insults, such as DNA damage and oxidative stress, inhibitory checkpoint regulators are activated at G1, S and G2 phases and stall the progression of cell cycle [7]. These regulators include DNA damage response genes (RAD1, HUS1, RAD9, ATM, ATR and p53) [8] and members of CDK inhibitor family, p16 (CDKN2A), p15 (CDKN2B), p21 (CDKN1A) and p27 (CDKN1B) [9]. In the subsequent M phase, mitotic checkpoint proteins of MAD and BUB families are responsible for a proper chromosomal segregation during cell division [10].

Studies performed on hepatic biopsies from chronic hepatitis C patients have shown an imbalance at G1/S checkpoint of cell cycle [11,12]. In fact, the report from Marshal et al showed a G1 arrest in hepatocytes characterized by over expression of CDK inhibitor p21. In our previous work [13] this arrest was further described by showing altered expression of tumor suppressor p53 and apoptotic proteins Caspase-3 and Bcl-2. Although these reports suggest modulations of cell cycle events in hepatocytes during the course of HCV-mediated disease progression, a detailed analysis of cell cycle regulators involved at other checkpoints still needs to be performed.

In order to determine the contribution of cell cycle genes in HCV-induced disease progression, we have studied the altered expression of cell cycle genes in HCV infected liver biopsy tissues. This might be considered as an initial step towards the prevention of chronic HCV disease progression into HCC. The mRNA expression of eighty four cell cycle genes were first examined in pooled RNA samples from patients with early or advanced HCV disease, and in normal liver RNA of an uninfected individual. This analysis identifies a list of differentially expressed genes involved at cell cycle checkpoints. The translational levels of four differentially expressed genes (p15, p27, KNTC1 and MAD2L1) were further examined. These genes were selected on the basis of their crucial roles at G1/S and mitotic checkpoints, in 38 chronic HCV infected tissues using immunohistochemistry or western blotting. The results showed significant association of p27 and KNTC1 with the progression of liver fibrosis.

## Methods

### Tissue Samples

After obtaining approval from the ethical review committee at the Aga Khan University, and informed consent from the patients, liver biopsy specimens were collected from thirty eight untreated chronic hepatitis C patients and six non-viral hepatitis patients. Each sample was apportioned into two pieces; one was frozen at -80°C and other was fixed in formalin. An experienced liver pathologist performed the histopathological grading and staging on formalin-fixed paraffin embedded tissue samples, employing Batts and Ludwig scoring system [14]. Fibrosis stage was F0, F1, F2, F3 and F4 in 5, 9, 6, 10 and 8 patients, respectively. Paraffin embedded tissues from individuals who had normal liver histology (n = 4) as determined by light microscopy, were also included as controls in the immunohistochemistry experiments.

### Sample Preparation and Quantitative RT-PCR

For transcriptional studies eight liver specimens were selected; four with F0-1, designated as early HCV disease and four with F3-4, designated as advanced HCV disease (Table 1a). Total RNA from frozen liver tissues was extracted in TRIzol reagent (Invitrogen, MD, USA). The extract was made DNA free by digestion with RNase-free DNase (Invitrogen, MD, USA) using 0.2 U/1 μg RNA and incubation at 37°C for 10 to 30 minutes followed by DNase inactivation at 75°C for 5 minutes. For PCR array experiments, human cell cycle RT^2 ^profiler PCR array (SuperArray Bioscience, MD) was used to simultaneously examine the mRNA levels of 89 cell cycle genes (list of genes is available at http://www.superarray.com), including five "housekeeping genes" in pooled RNA samples from early and advanced HCV group. The pooled samples were prepared by mixing equal amounts of liver tissue RNA from four patients of each group. Previous studies have described RNA pooling as an appropriate initial screening approach [15]. Given the limited availability of liver biopsies from normal liver patients, total RNA of normal, non-diseased liver purchased from Ambion (Palo Alto, CA) was used as a reference standard in these experiments. Reverse transcription was performed at 37°C for 1 hour with ReactionReady first strand cDNA Synthesis Kit (SuperArray Biosciences, MD) using 1 μg of total RNA. The synthesized cDNA was subjected to PCR amplification using RT^2 ^profiler PCR arrays according to manufacturers' instructions. The PCR conditions used were as follows; 95°C for 10 minutes followed by 40 cycles at 95°C for 30 seconds and 60°C for one minute. Each experiment was performed in duplicate or triplicate on iCycler iQ Real Time PCR system (BioRad Laboratories). Of the 5 housekeeping genes, GAPDH was used for normalization. Fold change in expression of different groups was calculated using ΔΔC^t ^method [16]. A p-value of ≤ 0.05 and a fold change of ≥ 2 in gene expression was taken as significant.

**Table 1:** Demographic features of the study patients

	Mean Age (years)	Gender (male/female)	Virus infection	*Stage of Fibrosis	*Grade of Inflammation
a) Patients included in PCR array experiments					
					
Early HCV (n = 4)	40 ± 6.2	2/2	HCV	0–1	0–1
Advanced HCV (n = 4)	53 ± 4.9	2/2	HCV	3–4	3–4

b) Patients included in Immunohistochemistry experiments					
					
Chronic HCV (n = 38)	42+9.3	18/20	HCV	0–4	1–4
Non-viral hepatitis (n = 6)	49 ± 2.1	2/4	None	1–4	1–3
Normal (n = 4)	26 ± 4.0	2/2	None	-	-

* Histopathological staging and grading of tissue samples were performed according to Batts Ludwig scoring system.

### Antibodies

The following antibodies were used for immunohistochemistry at the dilutions given in parentheses; Mouse monoclonal antibodies to p27 (DCS-72.F6, 1:50) and p15 (15P06, 1:25) were purchased from Abcam (Cambridge, UK). For western blotting monoclonal antibodies against MAD2L1 (17D10, 1:500) and KNTC1 (10H4, 1:1000) were purchased from Abcam (Cambridge, UK) and AbNova (Taiwan), respectively.

### Immunohistochemistry

For immunohistochemistry 4-micron thin sections of paraffin-embedded liver tissues (Table 1b) were deparaffinized and rehydrated using a xylene-alcohol sequential wash. The sections were then subjected to antigen retrieval by heating in 0.01 M citrate buffer (pH 6.0) using a pressure-cooker. EnVision Kit (Dako, Hamburg, Germany) was used for immunostaining essentially following the manufacturer's protocol. Briefly, endogenous peroxidase was blocked for 5 min in 0.03% H_2_O_2_. Sections washed in Tris-buffered saline with Tween 20 (TBST; 0.05 mol/l Tris and 0.15 mol/l NaCl, pH 7.6, 0.1% Tween20) were incubated with primary antibodies for 1 hour at room temperature. Thereafter, a horseradish peroxidase (HRP)-labelled polymer conjugated with a secondary antibody was applied. The staining was visualized with 3, 3'-diaminobenzidine tetrahydrochloride (DAB). Finally, the slides were counterstained with haematoxylin and mounted in DPEX for examination. The immunostained sections were examined using Nikon Eclipse E8000 and image analysis system, (Nikon, Japan). Quantification of positive hepatocytes was undertaken by two independent observers counting approximately 1000 hepatocytes at magnification 400× in five randomly selected fields. The positive hepatocytes were expressed as a percentage of the total cells counted in each case and values for subsequent analysis were obtained from the mean of the two independent observations.

### Protein Extraction and Western Blotting

Proteins were isolated from the organic phase of TRIzol extraction as described in manufacturer's protocol. Isolated proteins were heat denatured in electrophoresis sample buffer (62 mM Tris-Cl, pH 6.8, 0.2% glycerol, 2% SDS, 0.04% β-mercaptoethanol, 0.04% bromophenol blue) and loaded onto 12% SDS-polyacrylamide gel. After electrophoresis on the gel, separated protein bands were transferred onto a PVDF membrane (Amersham, Germany). Membranes were blocked with 5% Blotting Grade Blocker non-fat dry milk powder overnight at 4°C. The membrane was incubated with primary antibody diluted in 1× TBST (Tris-buffered saline, 0.1% Tween 20) on an orbital shaker, followed by three washings in TBST for 10 mins. After washing, the membrane was incubated with horse radish peroxidase-labelled anti-mouse IgG (1:10,000) for 1 h on an orbital shaker at room temperature. Membrane was washed with 1× TBST three times for 10 min on a shaker. The blot was developed with ECL Plus reagent (Amersham, Germany) according to manufacturer's instructions. Actin was included as internal loading control and detected by Anti-actin (Santa Cruz). Band intensities were measured using Image J software (NIH, USA).

### Statistical Analysis

Significant fold change in mRNA levels of each gene between the groups was tested using *t*-test as indicated by the manufacturer (SuperArray, MD). Analyses of immunostaining data were performed using the Statistical Package for Social Sciences (SPSS, 14.0). Association between the expression of p27, p15, KNTC1 and MAD2L1with histological feature fibrosis was analyzed using Johnkhere Terpestra test or Mann Whitney U-test. Correlations among the groups were evaluated by Spearman's rank correlation coefficient. A p-value of less than 0.05 was considered significant.

## Results

### Transcriptional Profile of Cell Cycle Pathway Genes

In order to assess the extent of modulation in cell cycle gene expression during chronic infection, PCR arrays of 84 cell cycle genes were performed in RNA samples separately pooled from early HCV, advanced HCV and commercial preparation of human normal liver RNA. The mean GAPDH threshold cycle (Ct) values for early HCV, advanced HCV and normal liver were 22.45 ± 0.5, 22.95 ± 0.42 and 22.0 ± 0.21 respectively. Comparisons of mRNA expressions of cell cycle genes, made between, 1) normal liver to early HCV and 2) early HCV to advanced HCV, revealed that fifteen (18%) of the 84 cell cycle genes were not detectable in any of the comparative groups, when Ct value greater than 35 was applied as a cutoff for significant expression.

The mRNA expression of 22 (31%) of the remaining 69 genes showed more than 2-fold change in early HCV versus normal liver (Table 2). Seven (32%) of these showed significant increase which include proliferation genes Mcm-2 and cyclin E1, DNA repair gene GADD45α (Growth arrest and DNA damage 45α), CDK inhibitor CDKN1B/p27 and G2-M phase regulatory genes KNTC1, DNM2 and CDC16 (see additional file 1).

**Table 2:** List of differentially expressed cell cycle genes >2 fold in early HCV relative to normal liver.

Gene Symbol	Average C_t _Early HCV	Average C_t _Normal	T-test p-value	Fold change
				
**Cell cycle progression/cell proliferation genes**				

MCM2	30.85	33.4	**0.05**	7.670
CDC2	32.5	34.15	0.36	4.79
MCM5	28.15	29.5	0.16	3.390
MCM3	28.0	29.1	0.11	3.100
CCNE1	33.6	34.9	**0.05**	4.76

				
**CDK Inhibitors**				

CDKN1A	27.5	28.2	0.258	2.24
CDKN1B	27.65	28.6	**0.034**	2.36

				
**G2/M checkpoint gene**				

GADD45A	27.15	29.8	**0.03**	9.000
DNM2	31.75	29.6	**0.005**	-2.65
KPNA2	31.3	29.25	0.169	-3.730

				
**Mitotic checkpoint genes**				

KNTC1	31.6	34.4	**0.04**	9.19
CKS1B	31.0	33.5	0.17	7.200
MAD2L1	32	34.55	0.077	7.07
CDC34	30.0	31.3	0.35	3.4
CDC16	32.6	30.8	**0.027**	-2.16

				
**DNA damage response genes**				

CHEK1	32.35	33.70	0.24	3.09
HUS1	29.65	27.05	0.06	-4.37

				
**Cell cycle Regulators**				

HERC5	29.3	33.7	0.07	20.393
CCNG2	29.85	32.3	0.11	6.400
RBBP8	31.7	30	0.108	-2.300
BCL2	29.65	31.60	0.052	3.10
UBE1	29.55	27.4	0.206	-3.500

The values present average C_t _values without normalization and fold differences observed in Real time PCR and p-values of T-test (values ≤ 0.05 are in bold).

In early versus advanced HCV comparison, 23 (33%) of the 69 genes showed more than 2-fold change (Table 3). Nine (29%) of these genes were significantly differentially expressed which include DNA damage response genes RPA3, RAD1, HUS1 and CDK inhibitors CDKN2B/p15, CDKN3 that were found up-regulated whereas tumor suppressor protein p53 and mitotic checkpoint protein MAD2L1 were found down regulated (see additional file 1).

**Table 3:** List of differentially expressed cell cycle genes >2 fold in advanced HCV relative to early HCV

Gene symbol	Average C_t _Adv. HCV	Average C_t _Early HCV	T-test p-value	Fold change
				
**Cell proliferation genes**				

CCND1	32.65	31.00	0.14	-2.22
CCND2	31.3	32.35	**0.05**	5.00
CDK2	32.1	30.3	0.6	-2.46

				
**CDK inhibitors**				

CDKN2B	32.4	33.50	**0.04**	3.03
CDKN3	34.20	32.73	**0.05**	5.46
CDKN1B	29.4	27.7	0.5	-2.3

				
**G2/M checkpoint gene**				

KPNA2	30.85	31.3	0.98	2.75
HERC5	33.05	29.3	0.217	-8.70

				
**DNA damage response genes**				

ATR	31.9	35	0.30	10.50
RAD1	30.4	32.8	**0.05**	6.46
HUS1	27.55	29.65	**0.05**	6.06
RAD17	28.45	30.35	0.057	5.27
RPA3	33.3	35.0	**0.01**	4.6
*TP53	30.80	27.03	**0.04**	-2.79

				
**Mitotic checkpoint regulators**				

MAD2L1	35	32	**0.04**	-5.6
CDC34	32.65	30.00	**0.025**	-4.4

				
**Cell cycle regulation**				

CCNH	32.85	35.0	0.5	5.00
CDK8	28.7	30.5	0.07	4.92
CCNG2	27.8	29.85	0.09	5.86
CKS2	30.7	32.15	0.23	3.86
CUL1	30.6	28.3	0.65	-3.48
TFDP2	28.2	30	0.08	4.92
CCNT1	28.6	31.15	0.08	4.59

The values present average C_t _values without normalization and fold differences observed in Real time PCR and p-values of T-test (values ≤ 0.05 are in bold).

### Protein Expression of CDK Inhibitors

**Figure 1:**
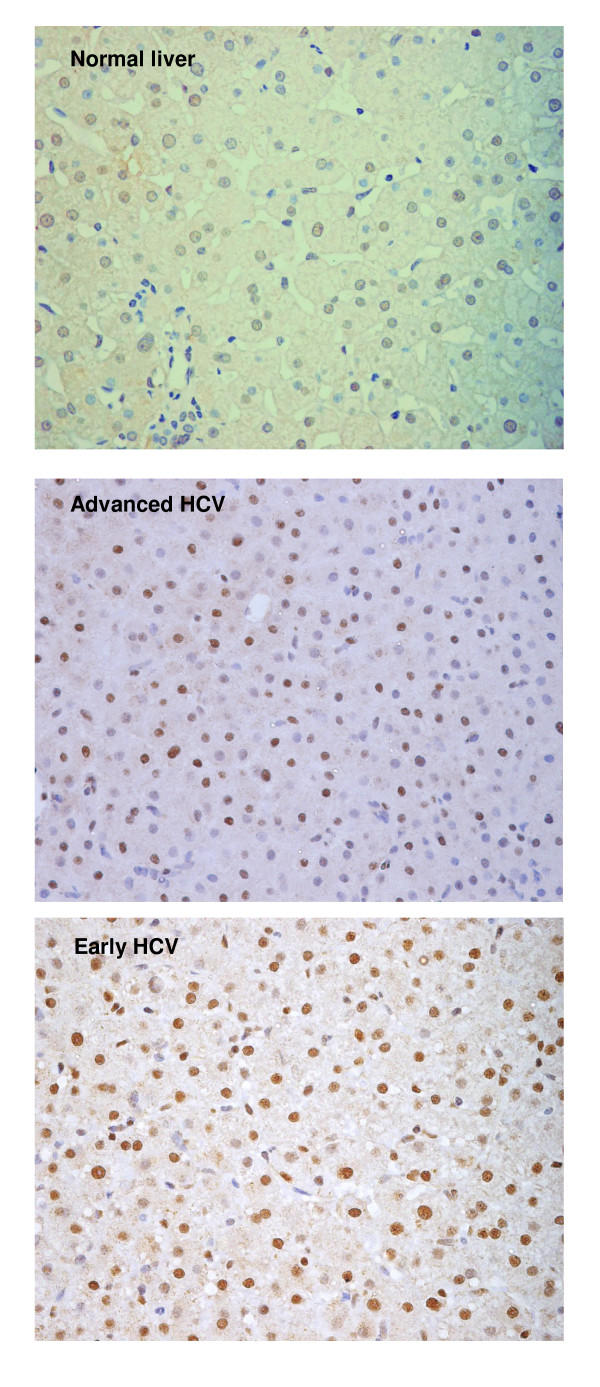
**Expression of CDK inhibitor p27**. Immunohistochemistry of p27 in normal liver and chronic HCV-infected liver with Early and Advanced HCV. Nuclear expression of the protein was observed in hepatocytes. Positive hepatocytes are stained brown (Mayer hematoxylin, magnification 400×).

**Figure 2:**
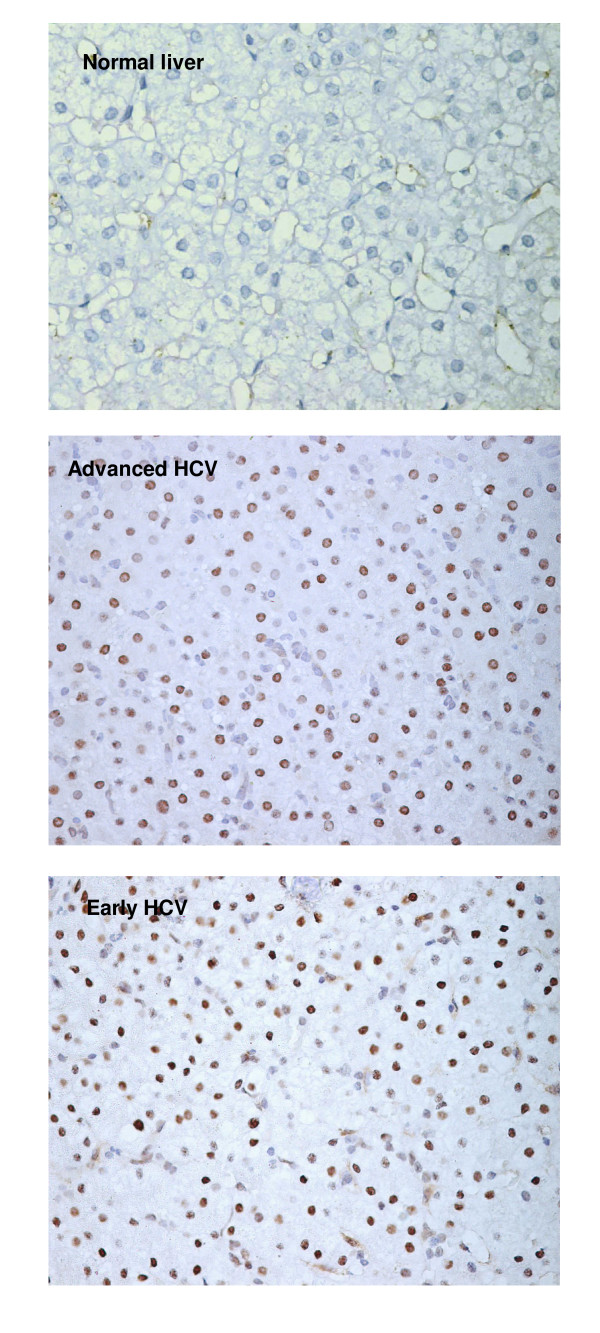
**Expression of CDK inhibitor p15**. Immunohistochemistry of p15 in normal liver and chronic HCV-infected liver with Early and Advanced HCV. Nuclear expression of the protein was observed in hepatocytes. Positive hepatocytes are stained brown (Mayer hematoxylin, magnification 400×).

**Figure 3:**
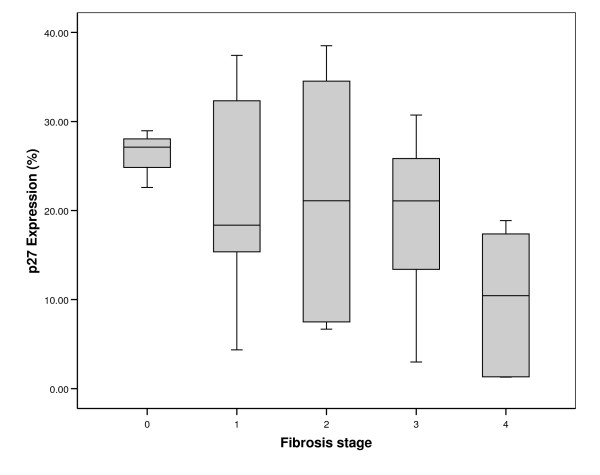
**Negative Association of p27 with fibrosis**. Box-plot graphic display of p27 expression in hepatocytes of chronic HCV patients grouped according to fibrosis stage. The horizontal bar in middle of the box represents the median, the box stretches between the 25^th ^and 75^th ^percentile, and the upper and lower lines extend to the range of the data. (Jonckeere-Terpstra test p = 0.04).

Experiments performed to evaluate the differential expression of cell cycle genes showed significant change in the expression of CDK inhibitors p27 and p15. Further investigation focusing on the expression of their corresponding proteins in paraffin embedded sections from 38 chronic hepatitis C patients (14 cases with F0 or F1, 6 cases with F2 and 18 cases with F3 or F4) resulted in varied profile. The p27 protein was found up-regulated in normal to early HCV while the p15 levels appeared to remain unaltered in early to advanced HCV. The immunostaining of each protein was localized in the nucleus of hepatocytes (Figure 1 &2) with few cases showing positivity in portal vein area as well. Expression of p27 was detected in the liver of 30/38 (78.9%) patients with chronic HCV infection. The percentage of p27 positive hepatocytes in early HCV (median = 22.6%, range = 4.3 – 37.4) was significantly higher as compared to normal liver (median = 4%, range = 1.3 – 6.3) (Mann-Whitney U test, p = 0.001) which was similar to the observations at transcriptional level. In advanced HCV the median and range of p27 expression were 17.3% and 3.0 – 32.0, respectively. The association of p27 expression with HCV-related F0 to F4 showed a significant decrease in p27 expression with the progression of fibrosis (Johnkhere Terpestra Test, p = 0.04, Figure 3). In non-viral hepatitis, p27 expression was rarely detected (see additional file 2).

Immunostaining of p15 was not evident in normal liver and non-viral hepatitis except in 1 case see additional file 2). p15 expression was nonetheless observed in 27/38 (71%) chronic HCV infected liver tissues. In contrast to what was observed at mRNA level a non-significant decrease in p15 expression was observed in early to advanced HCV comparison (median = 25%, range = 0–39 versus 21%, range = 13–37). Likewise, no significant association was found with the progression of fibrosis.

### Protein Expression of Mitotic Checkpoint Regulators

**Figure 4:**
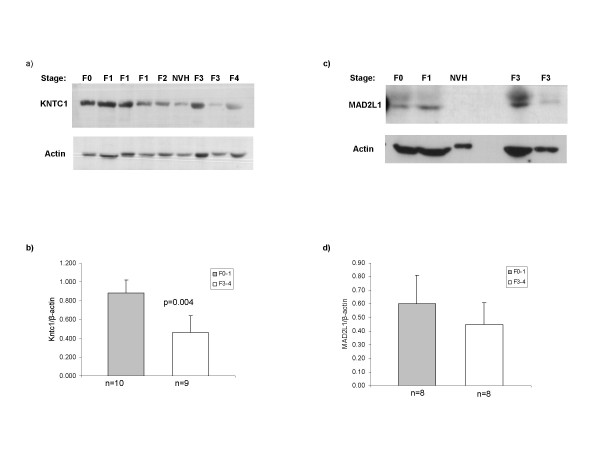
**Reduced expression of mitotic checkpoint genes in chronic HCV patients**. a & c) Western blotting for MAD2L1 and KNTC1 was done in total protein extracts prepared from biopsy specimens of chronic hepatitis C or Non-viral hepatitis patients (NVH). Following SDS-PAGE (12% gel) and transfer, the nitrocellulose membranes were probed with monoclonal antibodies to MAD2L1, which recognizes both phosphorylated and de-phosphorylated forms and KNTC1. b & d) Densitometry analysis of total MAD2L1 or KNTC1 expression normalized to their respective loading controls (β-actin) was done using Image J software. The number of samples analyzed in each group (n) is indicated below each column. The p-values at the top of the column compare early HCV to advanced HCV. Represented are mean ± S.D.

Two of the mitotic checkpoint proteins KNTC1 and MAD2L1 showed significant alterations in cell cycle array experiments. Owing to the limited amount of total proteins available from fresh frozen liver biopsies, the protein levels of both of these mitotic regulators were analyzed in 25 out of 38 chronic HCV patients and 4 non-viral hepatitis patients. Western blot analysis of KNTC1 detected its band in 20/25 chronic HCV patients and in 2/4 non-viral hepatitis patients (Figure 4a). A significant reduction (>2 fold, p < 0.01) in total KNTC1 levels was observed in advanced HCV as compared to early HCV (Figure 4b). The band of MAD2L1 was also detectable in 17/25 patients while in non-viral hepatitis it was not detectable (Figure 4c). Consistent with the observations at mRNA levels a slight decrease in its expression in advanced HCV group was observed but that was not found statistically significant (Figure 4d).

## Discussion

In the present study, an unbiased PCR array approach containing 84 cell cycle genes was applied on pooled RNA samples, to identify altered gene expression in HCV-associated liver disease. This analysis led to the identification of differentially expressed genes involved at cell cycle checkpoints, which may play a role in disease pathogenesis. Further investigation at protein level of four differentially expressed genes, selected on the basis of their crucial role in cell cycle regulation confirms the modulations in the expression of CDK inhibitor p27 and mitotic checkpoint genes MAD2L1 and KNTC1. It is important to note that these cell cycle proteins have not been implicated previously in chronic liver disease. Moreover, the association of p27 and KNTC1 expression with liver fibrosis suggests a role of these markers in the progression of liver disease.

Persistent inflammation and cell destruction during HCV infection has been suggested to induce proliferation of normally quiescent hepatocytes [17-19], which helps in restoring liver mass and maintaining hepatic function. Moreover, previous reports including our own have shown an arrested cell cycle in hepatocytes, during HCV infection [12,13]. Consistent with these data we found more than 3 fold increase in the mRNA levels of proliferation related genes Mcm-2, -3, -5, CDC2 and cyclin in early HCV group while the mRNA levels of cell cycle inhibitors p27, GADD45A, MAD2L1 and KNTC1 were also found to be significantly increased (Table 2). These transcriptional changes suggest a mixed population of cells in the infected livers of chronic hepatitis C some of which are in proliferating state while others in an arrested state. In advanced HCV, cell cycle inhibitors p27 and MAD2L1 were found down-regulated while p15/CDKN2B and CDKN3 were up-regulated. More than four fold increase was also observed in the mRNA levels of DNA damage response genes ATR, HUS1, RAD1, RPA3 and RAD17 (Table 3) suggesting increased DNA damage. These alterations were also accompanied by the down-regulation of tumor suppressor p53. Taken together these results are suggestive of deregulated cell cycle in chronic HCV infection that is considered as a consistent event in HCC [20,21].

Association of CDK inhibitor p21 with impaired liver regeneration and progressive liver fibrosis has been reported earlier [22]. In the current study, analysis at protein level by immunohistochemistry in HCV-infected hepatic biopsies showed a significant increase in the expression of p27 protein (Figure 1) that belongs to the same KIP family as p21. This protein is involved in inhibiting cell cycle progression at both G1 and S phases and is also defined as a tumor suppressor protein [23]. Interestingly, an inverse relationship between p27 protein expression and progression of liver fibrosis from F0 to F4 was found (Figure 3). It would be pertinent to mention here that mouse models of chronic injury induced-liver tumorgenesis have shown that loss of p27 in advanced stages of disease promotes tumor cell proliferation [24]. These observations signify the potential of p27 as a marker for prognostic implications in chronic HCV disease.

In contrast to p27, immunohistochemical evaluation of G1 phase inhibitor p15 revealed no significant difference in advanced HCV as compared to early HCV that conflicts with the changes observed at mRNA level. This inconsistency might have been due to decreased stability of p15 protein in advanced disease stages that increased the transcription through a regulatory feedback mechanism to maintain its levels. Another explanation for this inconsistency could be the use of pooled samples of whole liver lysates for mRNA expression while immunohistochemistry was done on individual formalin fixed paraffin embedded biopsies. Together, the expression of p27 and p15 in non-dividing hepatocytes might represent the population of cells which are arrested in cell cycle and not participating much in liver compensation, but are more active metabolically.

Defects in chromosomal segregation are a common feature of liver tumor cells suggesting a possible role of mitosis deregulation in the pathogenesis of HCC [25]. While examining the alterations of cell cycle genes in early and advanced HCV, we found altered expression of mitotic checkpoint genes, MAD2L1, KNTC1, CDC16 and CDC34. KNTC1, also known as "rough deal protein" (ROD) is part of a complex involved in elaborating an inhibitory signal due to improper chromosomal alignment during cell division [26], while member of Mad2 family, MAD2L1, participates in inhibiting Anaphase promoting complex (APC) [27] from ubiquitinating securin, whose degradation is a prerequisite for sister chromatid separation and mitosis. Considering the crucial roles of KNTC1 and MAD2L1 in chromosomal segregation, we analyzed their respective proteins by western blotting. Results showed increased levels of both of these proteins in early HCV that is suggestive of mitotic checkpoint activation or mitotic arrest in the liver cells. This is consistent with the down-regulation of CDC16, which is a component of APC and promote chromosomal segregation. Importantly, KNTC1 expression significantly decreases in advanced stages of HCV group as compared to early HCV while the expression of MAD2L1 showed a modest reduction in advanced HCV group (Figure 4). However, a possibility of error in assessing these changes could come about due to the fact that the expression of reference gene, β-actin also increases with the progression of liver fibrosis.

The role of KNTC1 in human cancers is not well documented, however, a single report describes mutation in this gene in colorectal carcinoma [28]. On the other hand, depletion or reduced expression of MAD2 gene has been reported in mammalian cells with loss of mitotic checkpoint and subsequent chromosomal instability [29,30]. Thus, low expression of these mitotic checkpoint regulators in advanced HCV might reflect loss of mitotic checkpoint control that could render cells to chromosomal instability. This hypothesis is also supported by a previous study which showed presence of near-aneuploidy DNA content in liver specimens from chronic hepatitis C patients [11].

The altered expression of cell cycle regulators observed in present study might be specific to hepatitis C as it was rarely observed in non-viral hepatitis patients. Nevertheless, the low number of control liver samples, hold back in achieving statistically significant differences between the two groups, with varying degrees of fibrosis.

## Conclusion

The analysis of cell cycle regulators showed altered expression of G1/S and M phase inhibitors in chronic HCV infection which either arrest or delays the progression through G1 and S phase as well as M phase, latter being not reported earlier in chronic hepatitis C patients. These cell cycle perturbations might be a consequence of increased DNA damage or persistent inflammation during viral infection that in turn could increase genetic instability and cell transformation. The information presented here laid down the basis for future studies to evaluate the activation of the identified cell cycle genes in chronic hepatitis C patients as well as HCC patients infected with HCV. These studies would be helpful to identify patients with high risk of cell transformation and HCC development.

## Competing interests

The authors declare that they have no competing interests.

## Authors' contributions

SS formulated the idea, conducted the experimental work and wrote the first draft of the manuscript. SH helped in the formulation of the study, supervised the acquisition of study samples, research protocol and the development of the manuscript. SA was involved in the analysis and interpretation of the data and was involved in drafting the manuscript. WJ was involved in the supervision of the study and acquisition of study samples. AS helped in the conception and design of the study and revised the manuscript critically for important intellectual content. All authors read and approved the final version of the manuscript.

## Pre-publication history

The pre-publication history for this paper can be accessed here:

http://www.biomedcentral.com/1471-2334/9/125/prepub

## Supplementary Material

Additional file 1 **Bar graph of differentially expressed cell cycle genes in early and advanced HCV**. Human Cell cycle RT-PCR-Array of pooled RNA samples from HCV infected liver specimens (early and advanced HCV) and normal liver RNA sample were performed. Bars represent fold differences in mRNA levels (> 2 fold, p < 0.05) of a particular gene when comparing a) Normal liver to Early HCV and b) Early HCV to Advanced HCV. Positive fold change values indicate that the transcript is up regulated, while negative values indicate that the transcript is down regulated. Click here for file

Additional file 2 Expression of CDK inhibitors in liver tissues with non-viral hepatitis. Click here for file
