# Supplementary material for: Modulations of cell cycle checkpoints during HCV associated disease
Source: BMC Infect Dis. 2009 Aug 10;9:125. doi: 10.1186/1471-2334-9-125 (PMC2739854; doi:10.1186/1471-2334-9-125)
Supplement: Additional file 1 — Bar graph of differentially expressed cell cycle genes in early and advanced HCV. Human Cell cycle RT-PCR-Array of pooled RNA samples from HCV infected liver specimens (early and advanced HCV) and normal liver RNA sample were performed. Bars represent fold differences in mRNA levels (> 2 fold, p < 0.05) of a particular gene when comparing a) Normal liver to Early HCV and b) Early HCV to Advanced HCV. Positive fold change values indicate that the transcript is up regulated, while negative values indicate that the transcript is down regulated. [file 1471-2334-9-125-S1.ppt]

## Slide 1
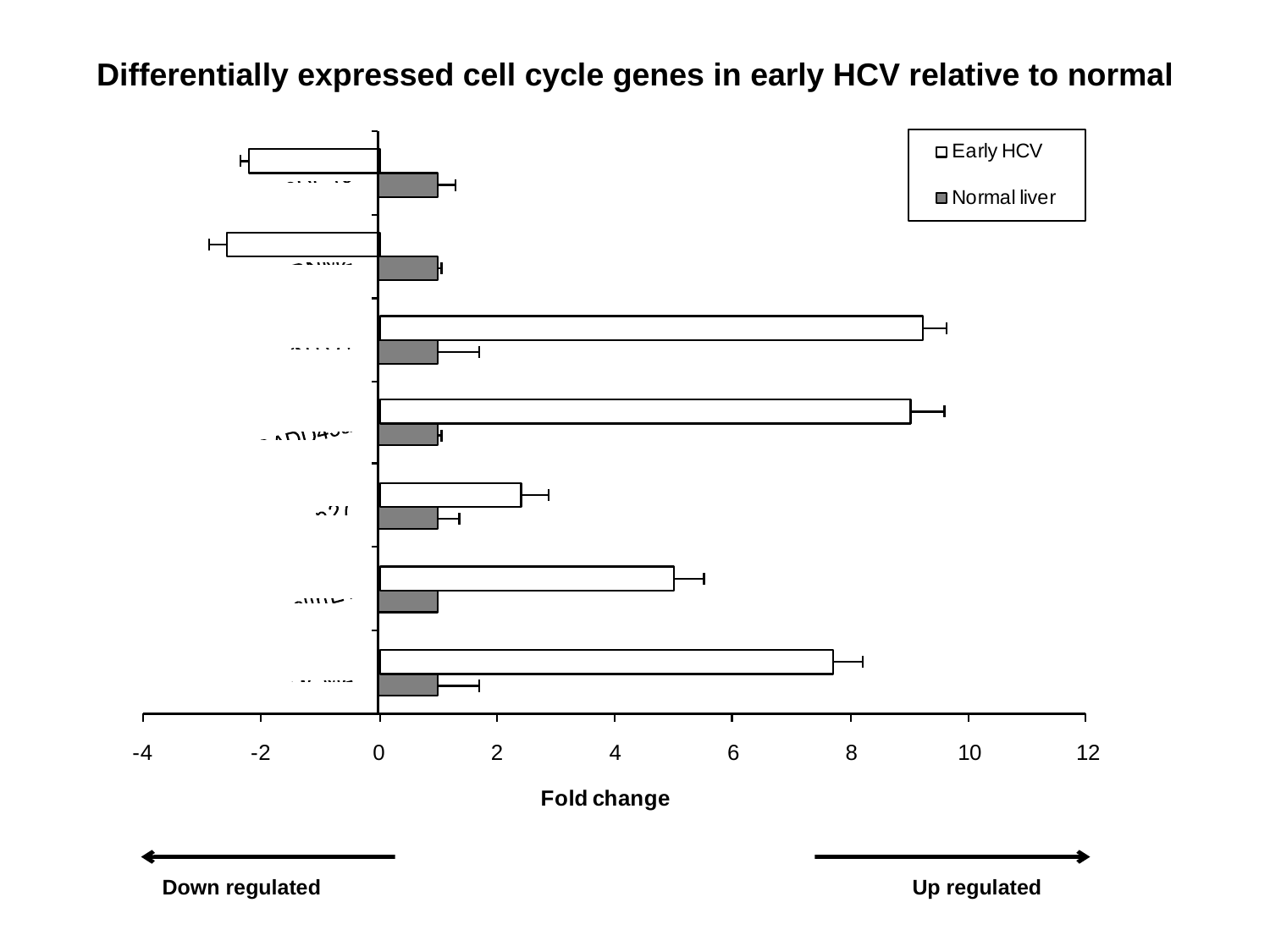

Differentially expressed cell cycle genes in early HCV relative to normal
Down regulated
Up regulated

## Slide 2
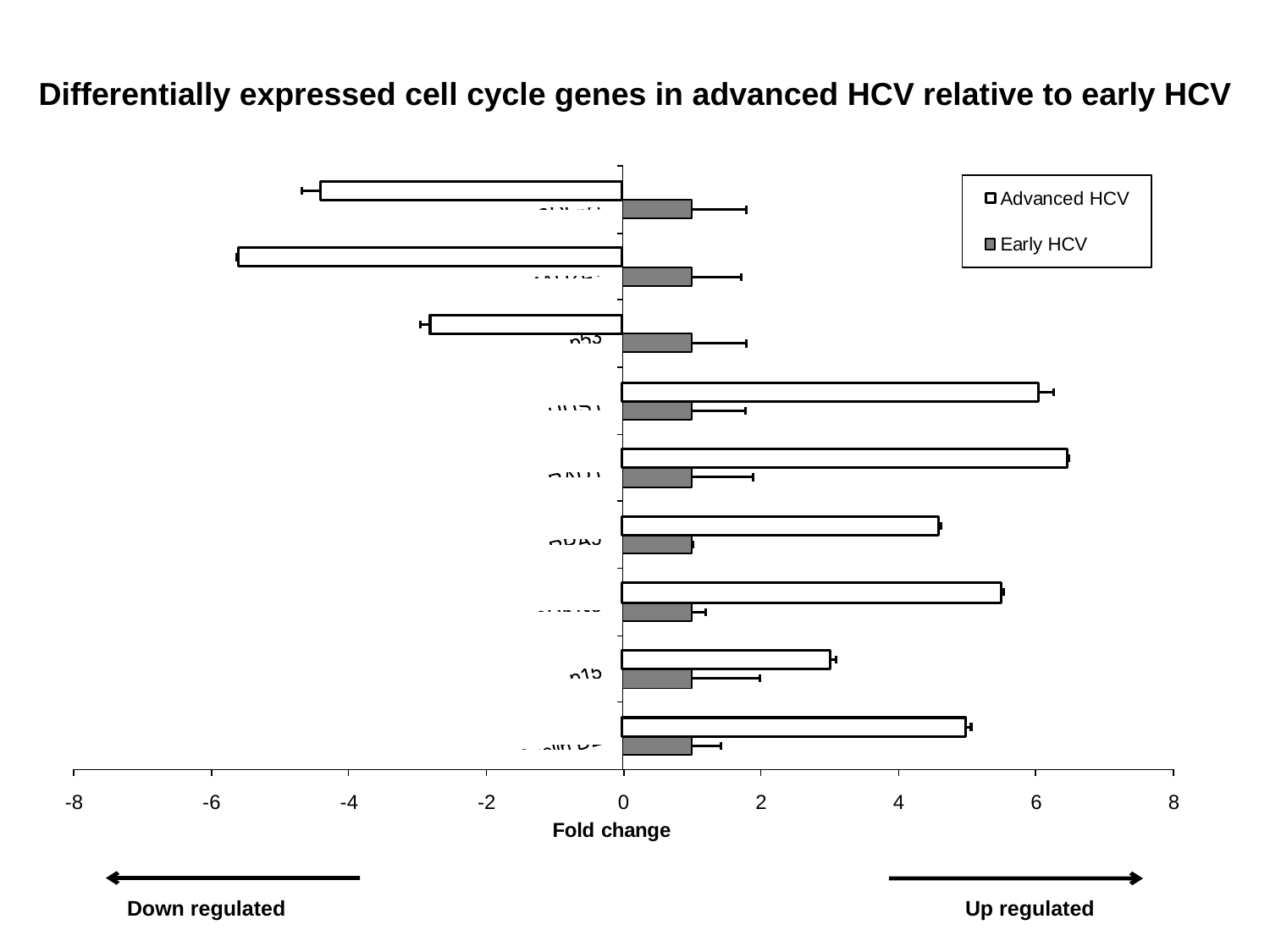

Differentially expressed cell cycle genes in advanced HCV relative to early HCV
Down regulated
Up regulated
