# Supplementary figures and images for: Modulations of cell cycle checkpoints during HCV associated disease
Source: BMC Infect Dis. 2009 Aug 10;9:125. doi: 10.1186/1471-2334-9-125 (PMC2739854; doi:10.1186/1471-2334-9-125)

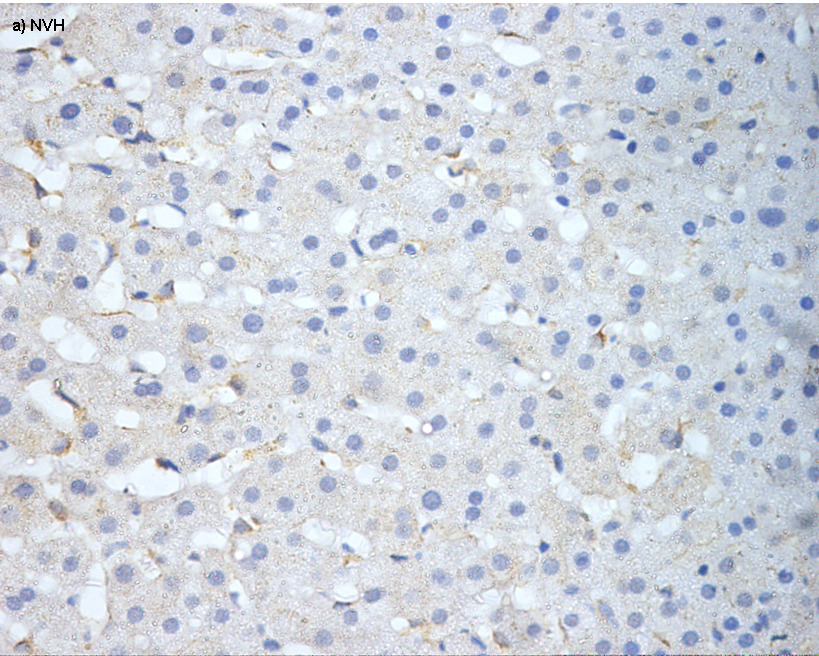


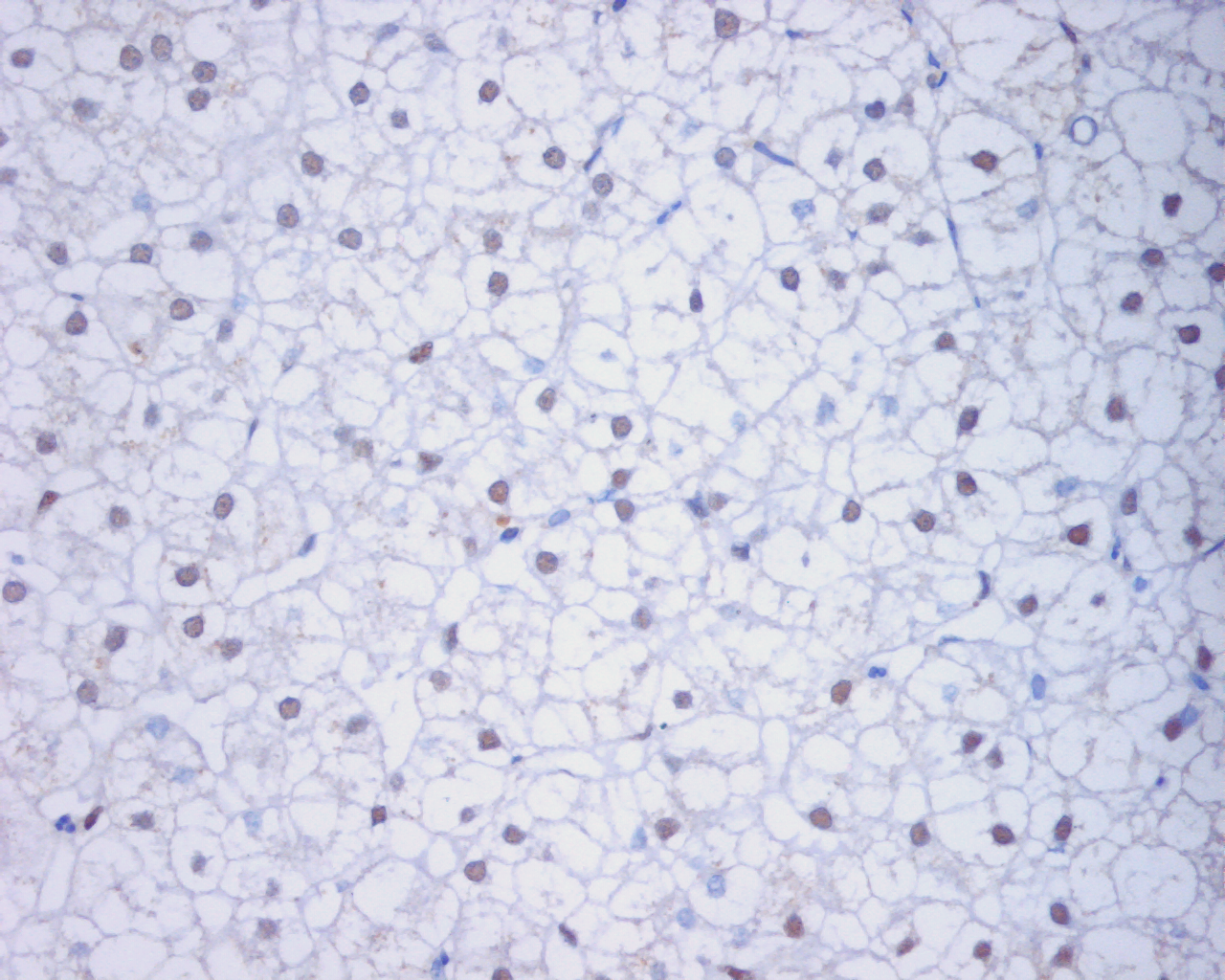


**b) NVH**

Supplement: Additional file 2 — Expression of CDK inhibitors in liver tissues with non-viral hepatitis. [file 1471-2334-9-125-S2.doc]
